# Molar Incisor Hypomineralization: Optimizing Treatment Protocols for Hypersensitivity: A Randomized Clinical Trial

**DOI:** 10.3390/dj12060186

**Published:** 2024-06-19

**Authors:** Elena Bardellini, Francesca Amadori, Laura Rosselli, Maria Luisa Garo, Alessandra Majorana, Giulio Conti

**Affiliations:** 1 Department of Medical and Surgical Specialties, Radiological Sciences and Public Health, School of Pediatric Dentistry, University of Brescia, Pl. Spedali Civili 1, 25123 Brescia, Italy; francesca.amadori@unibs.it (F.A.); l.rosselli@unibs.it (L.R.); alessandra.majorana@unibs.it (A.M.); 2 Mathsly Research, 25100 Brescia, Italy; marilu.garo@mathsly.it; 3 Department of Medicine e Surgery, School of Dentistry, University of Insubria, Via Ravasi 2, 21100 Varese, Italy; giulio.conti@uninsubria.it

**Keywords:** hypomineralization, hypersensitivity, dentin, laser, children

## Abstract

Dentin hypersensitivity (DH) is a common challenge in pediatric patients with molar incisor hypomineralization (MIH), stemming from enamel porosity or exposed dentin after enamel breakdown. This three-arm randomized controlled clinical trial aims to evaluate the effectiveness of three different desensitizing treatment protocols. The study was conducted on 39 children, aged 6–14 years old, with MIH and DH. Group A received casein phosphopeptide plus amorphous calcium phosphate fluoride (CPP-ACPF) mousse and sham light therapy, Group B received placebo mousse and photo-bio-modulation therapy (PMBT), and Group C received both CPP-ACPF mousse and PMBT. DH evaluation using a visual analogue scale was performed at multiple time points. Both CPP-ACPF mousse and PMBT individually demonstrated desensitizing effects on dental elements affected by MIH. While PMBT had a greater immediate effect, the combination of the two therapies proved most effective in reducing DH. The VAS scores were statistically lower in group C compared to groups A and B, both after the first session (*p* = 0.0001) and after 28 days (*p* = 0.0005). This study suggests promising avenues for managing DH in MIH patients, highlighting the potential of combined therapies, specifically CPP-ACPF mousse and PMBT, for enhanced clinical outcomes.

## 1. Introduction

Molar incisor hypomineralization (MIH) is a qualitative enamel defect that involves one or more permanent molars, often extending to the permanent incisors as well [1]. Enamel hypomineralization was first documented in the late 1980s, when several researchers reported congenital hypomineralization of the permanent teeth, particularly the first molars and incisors [1]. The European Academy of Paediatric Dentistry (EAPD) was the pioneering international scientific organization to undertake comprehensive studies on MIH and formulate a policy document [2]. Its latest clinical practice guidance was issued in 2021 [3]. Since the identification of MIH at the EAPD meeting in 2003, various assessment criteria have been observed across studies reporting its prevalence [4]. Accordingly, researchers employing the EAPD definition have indicated a higher prevalence of MIH (14.5%) in contrast to studies utilizing alternative diagnostic criteria (10.2%) [5,6]. MIH is believed to affect 14.2% of the population [5,6], impacting approximately 17.5 million children and adolescents globally [5,6], with no significant difference in prevalence between females and males [5,6]. Elfrink et al. [7], introduced the term “hypomineralized second primary molars” (HSPM) to describe a new entity, specifically MIH affecting second primary molars. Garot et al. [8] found a higher likelihood of MIH occurrence among individuals with HSPM, leading the authors to conclude, solely based on the presence/absence of defects, that HSPM serves as a prognostic indicator of MIH.

Several etiological hypotheses have been proposed for MIH. During the critical period, spanning from the 28th week of intrauterine life to the first few days of life, amelogenesis of the first permanent molars, permanent incisors, and second primary molars begins. Prenatal exposure to maternal smoking or illness, perinatal factors including premature or prolonged birth, low birth weight, cesarean-section delivery, and birth complications, as well as postnatal exposures like sixth disease (roseola), medications, or prolonged breastfeeding, have all been suggested as potential causes or associations with MIH. Nonetheless, a multifactorial pathogenesis with a potential genetic involvement appears probable [9,10].

The enamel affected by MIH is fragile and exhibits inferior mechanical properties [8,9], as it contains elevated levels of proteins that hinder the formation of hydroxyapatite crystals [8,9,10,11]. Despite ongoing research, a comprehensive understanding of the etiology of MIH remains elusive [2,3,4,5,6].

Clinically, affected teeth can vary in color shade from white to yellow or brown [12,13] and can show rapid wear, enamel loss, increased susceptibility to caries, loss of fillings and dentin hypersensitivity (DH) [12,13,14]. 

Tooth hypersensitivity to thermal or mechanical stimuli is a common clinical symptom in patients with MIH and often prompts individuals to seek dental care. Approximately 34% of teeth affected by MIH exhibit DH [15]. DH can manifest as spontaneous discomfort or pain after thermal or mechanical stimuli. Pediatric patients may experience anxiety, fear or behavioral problems during dental treatment [16], mostly because DH can persist even after local anesthesia. Children may also report pain when consuming hot and cold drink or meals or during toothbrushing, significantly impacting their quality of life. The exact cause of MIH sensitivity is not entirely clear [12]; it is likely that repeated stimuli might cause a subclinical pulp inflammatory response due to the enamel’s porosity [17]. Managing DH poses a significant challenge [15,16,17]. Several options are available, including using fluoride toothpaste, employing oral care products containing casein phosphopeptide-amorphous calcium phosphate (CPP-ACP), or casein phosphopeptide-amorphous calcium phosphate fluoride (CPP-ACPF) [18,19,20]. Additionally, topical sodium fluoride varnish application [19,21,22] with or without tricalcium phosphate and the use of devices such as ozone and low-level laser therapy (LLLT) [19,23,24] are among the options. While desensitizing agents seem to act by occluding dentinal tubules, LLLT appears to desensitize sensory nerves by blocking the transmission of pain stimuli from the dentinal tubules to the central nervous system [23] and ozone seems to promote remineralization [25].

A recent systematic review revealed that all studies investigating DH management reported improvement after treatment; however, none of these interventions can be strongly recommended due to moderate to high risk of bias, short follow-up periods, and small sample sizes [26]. 

Adding complexity to DH management, there is considerable uncertainty regarding treatment protocols in children, given the unique physiological and behavioral characteristics of this population compared to adults. With few studies focusing exclusively on this demographic, understanding effective treatment approaches for children with DH remains a challenge. This study aims to bridge this gap by comparing the effectiveness of different protocols in a pediatric cohort, offering valuable insights for clinical practice.

This randomized clinical trial compares three treatment protocols for reducing DH in children with MIH. Specifically, we investigated the effectiveness of CPP-ACPF (MI Paste Plus^®^, GC Italia S.r.l, Milan, Italy) mousse, photo-bio-modulation therapy (PBMT) using a diode laser (RAFFAELLO 980 BIO—Dental Medical Technologies—DMT S.r.l. Milan, Italy) and a combination both. The null hypothesis of this study was that there is no difference in DH reduction after CPP-ACPF or PBMT or CPP-ACPF and PBMT therapies.

## 2. Materials and Methods

### 2.1. Study Design

This involved a three-arm, randomized controlled clinical trial.

### 2.2. Ethical Consideration

This trial was submitted to and approved by the local Research Ethic Committee (ASST Spedali Civili of Brescia—code NP 4524—approval date 21 December 2020) and was registered on the clinical trials database (Registration number: NCT05705037). The study follows the SPIRIT (Standard Protocol Items: Recommendations for Interventional Trials) and CONSORT (Consolidated Standards of Reporting Trials) guidelines for randomized trials of nonpharmacological treatments [27]. Participants were included after their parents or caregivers had signed an informed consent form containing detailed information about the research. The study was conducted in accordance with the Declaration of Helsinki.

### 2.3. Examiner Calibration

Examiner calibration was conducted using 30 original photographs of teeth exhibiting MIH with varying degrees of severity, along with other developmental enamel defects. The assessments were based on the MIH index. After 15 days, both examiners reassessed all the photographs to evaluate inter- and intra-examiner agreement, which demonstrated high agreement levels (κ > 0.85) [28].

### 2.4. Sample Size and Selection of the Participants

**Figure 1:**
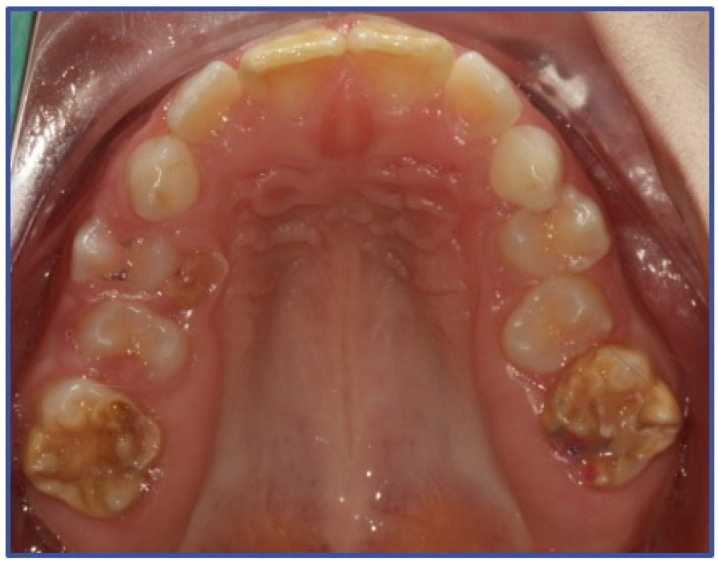
MIH- affected upper first permanent molars.

**Figure 2:**
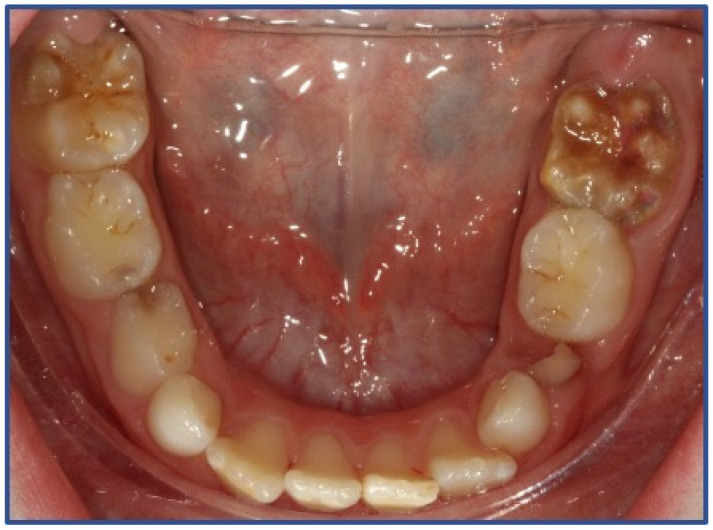
MIH- affected lower first permanent molars.

The sample was selected according to the following inclusion criteria: children aged 6–14 years, having at least one MIH-affected tooth and MIH-associated hypersensitivity. The diagnosis of MIH was carried out according to the European Academy of Paediatric Dentistry criteria [3] (Figure 1 and Figure 2). The assessment of hypersensitivity was performed using the Schiff Cold Air Sensitivity Scale (SCASS) [29], by applying a 1 s air jet at a distance of 1 cm from the occlusal surface of the tooth, with adjacent teeth shielded by the examiner’s fingers. Scoring was performed as follows: 0 = subject does not respond to air stimulus; 1 = subject responds to air stimulus but does not request discontinuation of stimulus; 2 = subject responds to air stimulus and requests discontinuation or moves away from stimulus; and 3 = subject responds to air stimulus, considers stimulus to be painful, and requests discontinuation of the stimulus. A score of 2 or 3 on the Schiff Cold Air Sensitivity Scale (SCASS) [29] was considered as a positive result, satisfying enrollment requirements. Exclusion criteria were allergy to milk proteins, chronic diseases, carious lesions or restorations on the sensitive teeth, other enamel defects (e.g., fluorosis, amelogenesis imperfecta, dentinogenesis imperfecta), presence of orthodontic appliance, recent anti-inflammatory or cortisone therapies, recent desensitizing treatments.

The sample size was calculated based on the existing literature and clinical experience, anticipating a reduction in hypersensitivity in 30% of untreated cases and 80% of treated cases. Considering an α error of 5%, study power of 80% and effect size 0.5, the sample size was determined to be 39 patients (at least 13 per group), using a goodness-of-fit test. The calculation accounted for the clustering effect in the analysis.

### 2.5. Random Allocation

The included patients were randomly allocated to three groups. Group A received the application of CPP-ACPF mousse (MI Paste Plus^®^, GC Italia S.r.l, Milan, Italy, LOT220803B) and sham light therapy; group B received the application of placebo mousse (Elmex Junior^®^Colgate-Palmolive Company, New York, NY, USA) and PMBT using a diode laser (RAFFAELLO 980 BIO—Dental Medical Technologies—DMT S.r.l, Milan, Italy); group C received both CPP-ACPF mousse and PMBT. The participants and their parents/caregivers were not aware of the group allocation. 

Randomization was achieved using a contingency number table obtained from www.random.org, (accessed on 30 June 2023) with the assignments preserved in sequentially numbered, sealed envelopes. Both the patients and operators (F.A. and L.R.) were unaware of the desensitizing treatment used, as both pastes were contained in similar receptacles, which hindered visual identification. The evaluators (E.B. and A.M.) who conducted the sensitivity assessment were blinded to the treatment groups. Data collection and analysis were performed by separate blinded operators (M.L.G. and G.C.).

### 2.6. Treatment Protocols

Treatment protocols were administered by two operators (F.A. and L.R.) who were previously trained and calibrated. 

The included patients were randomly allocated to three groups, each corresponding to a different treatment protocol. Each group underwent three desensitizing treatment sessions with a 7-day interval between each session. Each participant received a single intervention but could have more than one tooth treated. 

All the enrolled participants were provided with a toothbrush featuring soft bristles and a fluoride toothpaste, along with guidance on a non- cariogenic diet and oral hygiene practices. Immediately before the treatment, the teeth were thoroughly cleaned using a rotary brush and pumice stone paste. Each tooth subjected to treatment was isolated with a cotton roll.

The detailed protocol for each group is outlined below.

- Group A (CPP-ACPF mousse and sham light therapy)

CPP-ACPF mousse (MI Paste Plus^®^, GC Italia S.r.l, Milan, LOT220803B) was applied using a microbrush on the cervical buccal surface of each tooth for 5 min. Subsequently, it was evenly distributed for 20 s using a rubber cup coupled to a low-speed handpiece. Following this application, a sham therapy was conducted, using RAFFAELLO 980 BIO—Dental Medical Technologies—DMT S.r.l. Milan, Italy. Although the device was powered on, the hand piece remained inactive and sound effects were produced using a mobile phone. The laser device’s tip was positioned on two areas for each dental element, one in the center of the cervical region and the other in the middle third of the crown.

- Group B (placebo mousse and PBMT)

A placebo mousse (Elmex Junior^®^ Colgate-Palmolive Company- USA) was applied using a microbrush on the cervical buccal surface of each tooth for 5 min. It was then evenly distributed for 20 s using a rubber cup coupled to a low-speed handpiece. Subsequently, PBMT was administered, using RAFFAELLO 980 BIO—Dental Medical Technologies—DMT S.r.l. Milam, Italy. The laser device’s tip was positioned on two areas for each dental element, one located in the center of the cervical region and the other in the middle third of the crown. The parameters used were: wavelength 980 nm, power 4 W, irradiation area1 cm^2^, application time 15 s per 1 cm^2^, energy density 60 J/cm^2^.

- Group C (CPP-ACPF and PBMT)

CPP-ACPF mousse (MI Paste Plus^®^, GC Italia S.r.l, Milan, LOT220803B) was applied using a microbrush on the cervical buccal surface of each tooth for 5 min. Subsequently, it was evenly distributed for 20 s using a rubber cup coupled to a low-speed handpiece. Following this application, PBMT was administered, using RAFFAELLO 980 BIO—Dental Medical Technologies—DMT S.r.l. Milan, Italy The laser device’s tip was positioned on two areas for each dental element, one located in the center of the cervical region and the other in the middle third of the crown. The parameters used were: wavelength 980 nm, power 4 W, irradiation area 1 cm^2^, application time 15 s per 1 cm^2^, energy density 60 J/cm^2^.

### 2.7. Sensitivity Assessment

The assessment of DH was conducted with the Visual Analogue Scale (VAS) scale, using the Wong-Baker Faces Pain Rating Scale (WBFPRS) [30]. 

The VAS is a validated, subjective measure for acute and chronic pain, ranging from 0 indicating “no pain” and 10 indicating “worst pain” [30]. Since the study was conducted solely on pediatric patients, to record VAS, we used the WBFPRS, which is a pain assessment tool commonly used with children. This WBFPRS shows a series of faces ranging from a happy face at 0, or “no hurt”, to a crying face at 10, which represents “hurts like the worst pain imaginable”. Based on the faces and written descriptions, the children choose the face that best describes their level of pain. Children are asked to point to the face that best represents how they are feeling [30].

The sensitivity assessment was carried out following an evaporative stimuli, i.e., the application of an air blast from a syringe for three seconds at a pressure of 40 psi (20 ± 3 °C), perpendicularly to the buccal surface of the tooth, maintaining a distance of 0.5 cm. 

The evaluation of DH for each group was conducted at the following time points: prior to (T0) and following (T1) the first session, after the second session (T2) on the 7th day, following the third session (T3) on the 14th day and 4 weeks after starting, on the 28th day (T4). 

### 2.8. Statistical Analysis

Descriptive statistics are reported as mean and standard deviation for quantitative variables and frequency and percentage for quantitative variables. Fisher’s exact test and Kruskal–Wallis test with post hoc Dunn’s procedure were used to compare groups. Pre–post comparisons were made using the Friedman test. Statistical significance was set at 2% (*p* < 0.02) after Bonferroni correction. Data were analyzed at patient and tooth level. Statistical analyses were performed using STATA17 (StataCorp., College Station, TX, USA).

## 3. Results

**Figure 3:**
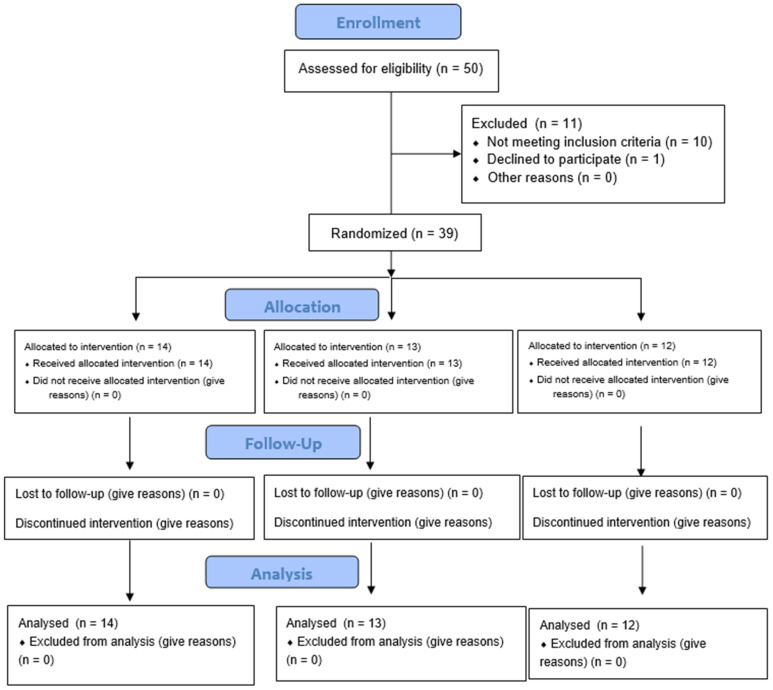
Study flow diagram.

Thirty-nine children (17 males and 22 females), aged 6–14 years old (mean age 9.56 ± 2.78), were enrolled (Figure 3), with 159 treated teeth. The demographic characteristics of the patients are shown in Table 1.

**Figure 4:**
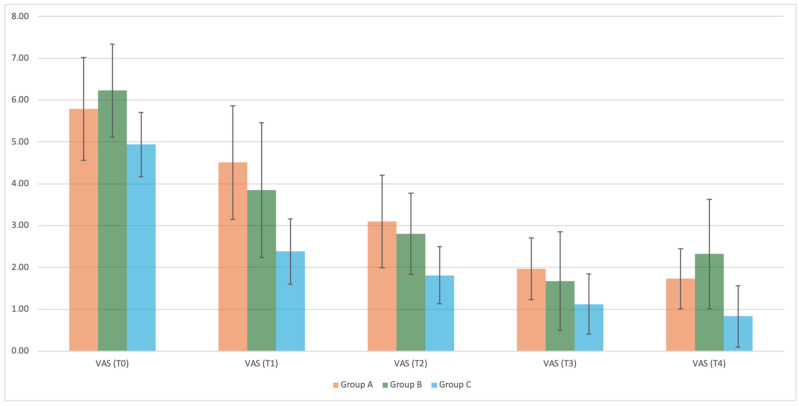
VAS mean of the groups at different evaluation time period (T0, T1, T2, T3,T4).

After the first session, VAS was statistically significantly lower in group C (2.38 ± 0.78) than in group B (3.85 ± 1.61, *p* = 0.0059) and group A (4.51 ± 1.36, *p* = 0.0001). VAS was lower in group C after seven and 28 days (7th day: 1.81 ± 0.68; 28th day: 0.83 ± 0.73) compared to Group B (7th day: 2.80 ± 0.97, *p* = 0.0055; 28th day: 2.32 ± 1.31, *p* = 0.0005) and Group A (7th day: 3.10 ± 1.11, *p* = 0.0011; 28th day: 1.73 ± 0.72, *p* = 0.0087) (Table 2) (Figure 4).

At the incisor level, VAS was higher after the first session and after 28 days in group B (T1: 4.25 ± 3.04; T4: 2.63 ± 2.33) than in group C (T1: 1.17 ± 0.94, *p* = 0.0023; T4: 0.42 ± 0.79, *p* = 0.0027) (Table 3).

VAS related to the molars showed statistically significantly higher VAS values in group B (3.91 ± 2.07) than in group C (2.63 ± 1.89, *p* = 0.0058), after the first session, at T1. This significant trend was also confirmed at T2, when group B (2.87 ± 1.60) had higher VAS scores than group C (2.05 ± 1.72, *p* = 0.0141). After 28 days, the VAS values were confirmed statistically significantly higher in group B (2.53 ± 1.61) than in group A (1.89 ± 1.47, *p* = 0.0012) and in group C (0.95 ± 1.41, *p* < 0.0001). (Table 4)

## 4. Discussion

DH is often present in patients with MIH. Enamel affected by MIH undergoes alterations including a reduction in mineral content, hardness, and elasticity modulus, accompanied by increased porosity [31,32,33]. These changes are associated with adverse clinical outcomes, such as post-eruptive breakdown, heightened risk of dental caries, and an increased need for restorative treatments [1,2,3,4]. Furthermore, research consistently shows elevated levels of DH in MIH-affected teeth [31,34].

The development of DH in MIH-affected teeth is thought to result from bacterial infiltration into dentinal tubules via porous enamel, leading to a sub-inflammatory response in the pulp tissue [35]. Various bacterial genera have been identified in the supragingival dental biofilm of MIH-affected teeth, including *Catonella*, *Fusobacterium*, *Campylobacter*, *Tannerella*, *Centipeda*, *Streptobacillus*, *Alloprevotella*, and *Selenomonas* [36]. Additionally, severe MIH cases with concurrent dental caries show elevated levels of *Lactobacillus*. 

According to Hernández et al. [36], the higher protein content of MIH-affected teeth could favor colonization by proteolytic microorganisms. Moreover, the over-representation of bacteria associated with endodontic infections and periodontal pathologies suggests that, in addition to promoting caries development, MIH could increase the risk of other oral diseases [36].

Mechanical and thermal stimuli can trigger discomfort in MIH-affected teeth [31,34], which may hinder effective oral hygiene practices and heighten the risk of dental caries [31,33]. This discomfort can negatively influence the oral health-related quality of life of those affected [16,31,37]. Additionally, DH in MIH-affected teeth can pose challenges in achieving effective local anesthesia, potentially compromising the success of required restorative treatments [38]. The difficulty in managing pain adequately may also lead to behavioral issues during clinical procedures [31].

Brännström’s hydrodynamic theory is widely acknowledged as the principal explanation for the mechanisms underlying the pain experienced in DH [39,40]. According to this theory, DH occurs due to the rapid movement of fluid within the dentinal tubules. When dentin is stimulated, fluid within the tubules moves towards and away from the pulp, activating baroreceptors in the pulp that transmit a sensation of pain to the central nervous system. The primary objective of DH treatment is to enhance quality of life by managing pain, achieved through either occlusion of dentinal tubules or desensitization of sensory nerves to block the transmission of nerve impulses [41,42]. This treatment approach aims to alleviate discomfort and improve patient well-being by addressing the underlying physiological processes responsible for DH symptoms.

The first desensitizing mechanism is exemplified by CPP-ACPF mousse. CPP is a casein-derived phosphoprotein able to bind and stabilize soluble ACP. Upon application, ACP breaks down into calcium and phosphate ions. The resulting state of dental mineral supersaturation promotes remineralization by precipitating as hydroxyapatite (HA), which occludes the dentinal tubules. The synergistic action of fluoride enhances this process, leading to the formation of fluorapatite (FA) and further contributing to the occlusion of the dentinal tubules [43,44].

The second desensitizing mechanism is found in photo-bio-modulation therapy (PBMT) [42]. PBMT induces alterations in nerve transmission originating from the dental pulp. This desensitizing effect of PBMT is achieved through the stimulation of nerve cells, specifically targeting the Na+/K+ pump located in the cell membrane. This stimulation alters the polarity of the cell membrane, increasing the amplitude of the membrane’s action potential and effectively blocking the transmission of painful stimuli [42,45]. Furthermore, PBMT’s regenerative properties contribute to increased metabolic activity in odontoblast-like cells, facilitating the enhanced production of tertiary dentin. This process effectively obliterates the dentinal tubules, with treatment protocols typically involving energy densities ranging from 3 to 10 J/cm^2^ [42,45,46,47,48,49]. Moreover, many studies demonstrated that PBMT also seems to reduce periodontal pathogens [50,51].

Despite the reported effectiveness demonstrated in numerous in vitro and in vivo studies, controversies persist regarding desensitizing protocols, application methods, and overall clinical effectiveness [52]. Therefore, the aim of this study was to conduct a comparative analysis of the use of CPP-ACPF, PBMT, or a combination of both techniques. 

A similar trial was conducted by Guanipa Ortiz et al. [44] on adult patients in 2019. The authors analyzed 24 patients aged 18–50 years and concluded that the combination of CPP-ACPF and PBM showed the largest reduction in DH. Given the lack of other studies assessing the efficacy of this specific combined treatment, our study focused on a larger, exclusively pediatric sample, with the intention of evaluating the efficacy of this protocol in children. Both trials used the VAS scale as a method of evaluating DH, allowing for a direct comparison of the effectiveness of the different protocols.

According to our results, the null hypothesis must also be rejected. Significant differences in DH outcomes were found among the three treatment groups.

Both CPP-ACPF mousse and PBMT, as well as their combination, exhibited a desensitizing effect, resulting in a statistically significant reduction in VAS scores following their application. PBMT demonstrated a more immediate effect compared to CPP-ACPF mousse; however, the combination of these two therapies proved to be the most effective in terms of desensitization. The combined approach synergistically enhanced the desensitizing outcomes, highlighting the potential for improved efficacy when utilizing both treatments concurrently.

When these two therapies are combined, their mechanisms of action may synergistically enhance the overall desensitizing effect. The regenerative properties of PBMT can contribute to the formation of tertiary dentin, which, when combined with the dentinal tubule occlusion facilitated by CPP-ACPF, offers a more comprehensive and robust defense against pain sensation. Moreover, the immediate effect of PBMT can complement the longer-term occlusive effects of CPP-ACPF, resulting in a more well-rounded and immediate reduction of DH. This integrated approach harnesses the unique strengths of each therapy to deliver enhanced and sustained relief from dental sensitivity associated with MIH.

Combined therapies for MIH-related DH in children appear to be promising. A recent study [53] investigated the effectiveness of erbium-doped yttrium garnet (Er:YAG) laser and GLUMA desensitizer for DH in teeth affected by MIH in pediatric patients. Zhao et al. concluded that the combination therapy of Er:YAG laser and GLUMA desensitizer had greater desensitizing effects than the use of the individual therapies and also positively impacted the oral health-related quality of life. Fossati et al. [54] recently proposed a study design to determine whether PBMT enhances the results of treatment with glass ionomer sealant on molars with MIH that present sensitivity. The study is ongoing and the results are not yet available.

While the present study provides valuable insights into the effectiveness of combined treatment protocols for DH in a wide pediatric population, a few constraints should be considered. 

Firstly, one such drawback might be the use of the WBFPRS for evaluating DH in children. Quantifying sensitivity in this age group is challenging, because responses to stimuli depend on the individual and this should be taken into account when interpreting the results [15,19]. In this study, we used the SCASS scale solely for enrolling children who complained of DH. To evaluate the response to the treatment, we chose the VAS scale using the WBFPRS. Another usable scale could be the FLACC (face, legs, activity, cry, consolability), which assesses the patient’s reaction to a nociceptive stimulus. However, we opted to restrict the assessment to a single scale to streamline the study. This highlights that the use of WBFPRS has been debated, due to concerns about its subjective nature in children who may confound pain measurements with non-nociceptive states. However, a recent study has shown that the WBFPRS exhibits a moderate correlation with the VAS and it is not mistaken for fear among school-aged patients [55]. 

Secondly, patients were enrolled based solely on the presence of DH, without considering the severity of MIH. This approach might result in a group that is not entirely homogeneous in terms of dental structure, potentially introducing variability in treatment responses. 

Additionally, the age range of participants was quite broad, spanning from 6 to 14 years. This wide range could lead to differing responses to treatment due to variations in the degree of molar mineralization and developmental stages. Younger children might have less mature dentin and enamel, which could affect the treatment outcomes differently compared to older children. 

Moreover, the observation period for this study was relatively short, at only 28 days. While this timeframe allowed us to assess the initial effectiveness of the treatments, a longer follow-up period would be beneficial to evaluate the long-term stability and effectiveness of the desensitizing treatments.

Despite these limitations, our study provides a foundational understanding of the relative effectiveness of combined DH treatments in pediatric patients and highlights the need for further research. Future studies with larger, more homogeneous samples, narrower age ranges, and extended follow-up periods will help to confirm and expand upon our findings.

## 5. Conclusions

DH poses a significant challenge for pediatric individuals with MIH.

The findings of this pediatric-focused study shed light on the potential avenues for effectively managing DH in children with MIH, emphasizing the promising role of combined therapies for enhanced clinical outcomes. Further research is warranted to substantiate this hypothesis and explore the potential synergies between PBMT and CPP-ACPF. It will be crucial to focus on finding the optimal pairing between a specific laser therapy and a desensitizing agent in the future. Optimizing the combined application of these therapies holds the potential to significantly improve clinical outcomes in the management of DH in pediatric patients with MIH.

## Figures and Tables

**Table 1:** Demographic characteristics of the patients.

	Total	Group A (CPP-ACPF and Sham Light Therapy)	Group B (PBM and Placebo Mousse)	Group C (CPP-ACPF and PBM)	*p*
**Gender**					
Female n (%)	22 (56.41%)	8 (57.14%)	8 (61.54%)	6 (50.00%)	
Male n (%)	17 (43.59%)	6 (42.86%)	5 (38.46%)	6 (50.00%)	0.920
**Age (yrs)**					
Mean (SD)	9.56 (2.78)	9.17 (2.59)	9.85 (2.67)	9.64 (3.18)	0.773
Median (min–max)	9 (6–14)	8 (6–14)	9 (6–14)	9 (6–14)	
**Teeth**					
Incisors n (%)	36	8 (22.22%)	16 (44.44%)	12 (33.33%)	
Molars n (%)	123	44 (35.77%)	38 (30.89%)	41 (33.33%)	

**Table 2:** VAS mean and standard deviation of the groups at the different evaluation time periods (T0, T1, T2, T3, T4).

	Total	Group A (CPP-ACPF and Sham Light Therapy)	Group B (PBM and Placebo Mousse)	Group C (CPP-ACPF and PBM)	*p*
VAS (T0)	5.68 (1.16)	5.79 (1.23)	6.23 (1.11)	4.94 (0.77)	0.020
VAS (T1)	3.63 (1.56)	4.51 (1.36)	3.85 (1.61)	2.38 (0.78)	0.001 ^a^
VAS (T2)	2.60 (1.07)	3.10 (1.11)	2.80 (0.97)	1.81 (0.68)	0.005 ^b^
VAS (T3)	1.61 (0.95)	1.97 (0.74)	1.67 (1.18)	1.12 (0.72)	0.083
VAS (T4)	1.65 (1.11)	1.73 (0.72)	2.32 (1.31)	0.83 (0.73)	0.004 ^c^
*p*-value		<0.0001 ^§^	<0.0001 ^§^	<0.0001 ^§^	

(^a^) PBM vs. CPP-ACPF and PBM (*p* = 0.0059) and CPP-ACPF vs. CPP-ACPF and PBM (*p* = 0.0001), (^b^) PBM vs. CPP-ACPF and PBM (*p* = 0.0055) and CPP-ACPF vs. CPP-ACPF and PBM (*p* = 0.0011), (^c^) PBM vs. CPP-ACPF and PBM (*p* = 0.0005) and CPP-ACPF vs. CPP-ACPF and PBM (*p* = 0.0087), ^§^ *p* < 0.01 two-by-two comparisons between T0 and T1, T2, T3 and T4.

**Table 3:** VAS mean and standard deviation of the incisor teeth at the different evaluation time periods (T0, T1, T2, T3, T4).

	Total	Group A (CPP-ACPF and Sham Light Therapy)	Group B (PBM and Placebo Mousse)	Group C (CPP-ACPF and PBM)	*p*
Incisor teeth (n)	36	8	16	12	
VAS (T0)	3.44 (2.53)	2.50 (0.93)	4.94 (2.95)	2.08 (1.44)	0.025
VAS (T1)	2.64 (2.55)	1.63 (0.74)	4.25 (3.04)	1.17 (0.94)	0.015 ^a^
VAS (T2)	1.72 (1.53)	0.88 (0.69)	2.81 (1.74)	0.83 (0.64)	0.165
VAS (T3)	1.25 (0.75)	0.25 (0.17)	2.25 (1.88)	0.58 (0.51)	0.036
VAS (T4)	1.36 (0.99)	0.25 (0.17)	2.63 (1.98)	0.42 (0.31)	0.003 ^b^
*p*-value		<0.0001 ^§^	<0.0001 ^§^	<0.0001 ^§^	

(^a^) PBM vs. CPP-ACPF and PBM (*p* = 0.0023), (^b^) PBM vs. CPP-ACPF and PBM (*p* = 0.0027) and PBM vs. CPP-ACPF and PBM (*p* = 0.0023), ^§^ *p* < 0.01 two-by-two comparisons between T0 and T1, T2, T3 and T4.

**Table 4:** VAS mean and standard deviation of the molar teeth at the different evaluation time periods (T0, T1, T2, T3, T4).

	Total	Group A (CPP-ACPF and Sham Light Therapy)	Group B (PBM and Placebo Mousse)	Group C (CPP-ACPF and PBM)	*p*
Molar teeth (n)	123	44	38	41	
VAS (T0)	6.07 (2.10)	6.16 (1.90)	6.53 (1.93)	5.56 (2.37)	0.145
VAS (T1)	3.76 (2.12)	4.68 (1.91)	3.92 (2.07)	2.63 (1.89)	<0.0001 ^a^
VAS (T2)	2.75 (1.78)	3.30 (1.80)	2.87 (1.60)	2.05 (1.72)	0.006 ^b^
VAS (T3)	1.77 (1.60)	2.16 (1.49)	1.84 (1.65)	1.29 (1.18)	0.021
VAS (T4)	1.77 (1.61)	1.89 (1.47)	2.53 (1.61)	0.95 (0.87)	< 0.0001 ^c^
*p*-value		<0.0001 ^§^	<0.0001 ^§^	<0.0001 ^§^	

(^a^) PBM vs. CPP-ACPF and PBM (*p* = 0.0058), (^b^) PBM vs. CPP-ACPF and PBM (*p* = 0.0141) and CPP-ACPF and PBM vs. CPP-ACPF (*p* = 0.0009), (^c^) PBM vs. CPP-ACPF and PBM (*p* = 0.0000) and CPP-ACPF and PBM vs. CPP-ACPF (*p* = 0.0012), ^§^ *p* < 0.01 two-by-two comparisons between T0 and T1, T2, T3 and T4.

## Data Availability

The data that support the findings of this study are available from the corresponding Author, (E.B.) upon reasonable request.
